# A comparison of the brief international cognitive assessment for multiple sclerosis and the brief repeatable battery in multiple sclerosis patients

**DOI:** 10.1186/s12883-015-0460-8

**Published:** 2015-10-15

**Authors:** Claudia Niccolai, Emilio Portaccio, Benedetta Goretti, Bahia Hakiki, Marta Giannini, Luisa Pastò, Isabella Righini, Monica Falautano, Eleonora Minacapelli, Vittorio Martinelli, Chiara Incerti, Ugo Nocentini, Giuseppe Fenu, Eleonora Cocco, Maria Giovanna Marrosu, Elisa Garofalo, Ferdinando Ivano Ambra, Maurizio Maddestra, Marilena Consalvo, Rosa Gemma Viterbo, Maria Trojano, Nunzia Alessandra Losignore, Giovanni Bosco Zimatore, Erika Pietrolongo, Alessandra Lugaresi, Lorena Pippolo, Marco Roscio, Angelo Ghezzi, Debora Castellano, Sergio Stecchi, Maria Pia Amato

**Affiliations:** Department of NEUROFARBA, University of Florence, Largo Brambilla 3, Florence, 50134 Italy; Don Gnocchi Foundation, Florence, Italy; IRCCS Hospital San Raffaele, Milan, Italy; University of Rome “Tor Vergata” c/o I.R.C.C.S. “Santa Lucia” Foundation, Rome, Italy; University of Cagliari, Cagliari, Italy; Hospital of Colli Monaldi Cotugno CTO, Naples, Italy; Hospital of Lanciano, Lanciano, CH Italy; University of Bari, Bari, Italy; Hospital of Barletta, Barletta, Italy; Department of Neuroscience, Imaging and Clinical Sciences, University “G. d’Annunzio”, Chieti, Italy; MS Centre, Hospital of Gallarate, Gallarate, Italy; IRCCS Institute of Neurological Sciences, Bologna, Italy

**Keywords:** Multiple sclerosis, Assessment tool, Cognitive impairment, Brief international cognitive assessment for multiple sclerosis

## Abstract

**Background:**

Recently, a Brief International Cognitive Assessment for Multiple Sclerosis (BICAMS) has been developed as an international and standardized brief cognitive test, which is easily performed in everyday clinical practice for neuropsychological assessment in multiple sclerosis (MS). However, we need to gather more information about this tool compared to other neuropsychological batteries. The aim of our study is to compare the performance of BICAMS and Brief Repeatable Battery (BRB) in MS subjects.

**Methods:**

Tests of the BRB and BICAMS were administered to MS patients recruited from 11 Italian MS centres. Cognitive impairment (CI) was defined as the failure on at least two tests (scores below the fifth percentile) on the BRB and as the failure on at least one test of the BICAMS. The agreement between the performances on the two batteries was assessed through Cohen’s K statistic. Finally we calculated the effects sizes for each test of the two batteries using Cohen’s d.

**Results:**

The two batteries were administered to 192 MS patients (142 women, 50 men; mean age 41.4 ± 10.8 years, mean education 12.3 ± 3.5 years). Mean scores of patients were lower compared to those of healthy subjects in all the cognitive measures examined. Forty-six MS patients were identified as impaired and 48 as unimpaired on both of the batteries, when the Symbol Digit Modalities Test (SDMT) was included in the analysis. Cohen’s K statistic was 0.46 which corresponds to a moderate accord. If the SDMT was excluded from the BRB, 37 MS patients were identified as impaired and 57 as unimpaired on both of the batteries. Cohen’s K statistic was 0.3 which corresponds to a poor accord. The SDMT, the Paced Auditory Serial Addition Test (PASAT) 3 and 2 yielded higher d values (SDMT 0.83, PASAT 3 0.65, PASAT 2 0.84).

**Conclusions:**

This study confirms the feasibility of BICAMS in everyday clinical practice for the identification of CI and highlights the good psychometric properties of the SDMT.

## Background

Cognitive impairment (CI) affects about 40–60 % of multiple sclerosis (MS) subjects [1]. It involves all the disease subtypes and it can be documented from the very beginning of the disease [1, 2]. Once established, it tends to progress over time, sometimes independently from the accumulation of physical disability [1]. Deficits of complex attention, information processing speed, episodic memory and executive functioning are prominent, whereas language and general intelligence are usually spared [3]. Also independently of physical disability, CI can have an important negative impact on patient performance in everyday activities, employment, social and recreational activities [1]. For this purpose, assessment of MS-related CI is strongly recommended. The most commonly used instrument to estimate cognitive dysfunction in MS patients, both for clinical practice and research purposes, is the Brief Repeatable Battery (BRB), that includes cognitive domains most frequently affected [4]. Normative values in the Italian population are available [5]. Despite its good psychometric properties, the implementation in clinical practice is limited by its time-consuming nature (about 45 min) and the need of administration and interpretation by experienced neuropsychologists. Therefore, there has been considerable effort over the past decade to streamline the neuropsychological assessment in MS, by developing brief assessment tools that can be incorporated in everyday patient assessment. In particular, recently, a Brief International Cognitive Assessment for MS (BICAMS) has been recommended as an international, validated and standardized brief cognitive test [6]. It is easily performed in everyday clinical practice as it can be completed in 15 min and can be administered by health care professionals who are not cognitive specialists. No special equipment (beyond pen, paper and stopwatch) is required [6, 7]. Translation and validation of the BICAMS is on going in several countries. It has been recently validated in the American [7], Czech [8], Iranian [9] and Italian populations [10]. We can consider BICAMS as a brief, practical and universal assessment tool for CI in MS subjects. However, little is known on its performance in comparison to other neuropsychological batteries. For this purpose, the aim of our study is to compare the performance of BICAMS and BRB as screening tools for cognitive impairment in MS patients [5, 10].

## Methods

A total of 192 MS patients (142 women; 50 men), among those consecutively admitted to some of the major Italian MS centres (Bari, Barletta, Bologna, Cagliari, Chieti, Florence, Gallarate, Lanciano, Milan, Naples and Rome), were recruited. Inclusion criteria were diagnosis of relapsing-remitting (RR) MS [11] and age >18 years. Inclusion was restricted to RRMS subjects in order to avoid heterogeneity of cognitive profile due to MS course. Exclusion criteria were presence of current or past neurological disorder other than MS, major psychiatric illness, history of learning disability, serious head trauma, alcohol or drug abuse and relapse and/or corticosteroid use within 4 weeks preceding assessment. All the subjects had adequate vision and hearing to undergo the tests. All the participants in the study provided their informed consent and the study was approved by the ethic committee of the University of Florence.

### Neuropsychological test procedures

At each site, patients were examined by the same neuropsychologist, who had participated to a common training session, in order to ensure uniform administration, data recording and scoring procedures. Tests of the BRB and BICAMS were administered in a standardized manner, during daytime, in a quiet room, and in a fixed order. We first administered the BRB and subsequently the BICAMS, in different sessions, within 1 week. The SDMT [4] was given only once. We used the validated Italian translation of both batteries [5, 10]. The BRB incorporates tests of verbal memory acquisition and delayed recall (Selective Reminding Test- SRT), visual memory acquisition and delayed recall (10/36 Spatial Recall Test-SPART), attention, concentration and speed of information processing (Paced Auditory Serial Addition Test –PASAT; SDMT) and verbal fluency on semantic stimulus (Word List Generation-WLG) [4]. The administration of the whole BRB battery takes about 45 min. The BICAMS includes the SDMT [4], California Verbal Learning Test, second edition (CVLT-II) first five trials, for assessing verbal memory [12] and Brief Visuospatial Memory Test-Revised (BVMT-R) first three recall trials, for visual-spatial memory [13]. Administration of the whole battery takes about 15 min.

### Statistical analysis

Group comparisons were assessed through Student’s t test, Mann–Whitney U test and χ^2^ test, as appropriate. CI was defined as the failure on at least two tests (scores below the fifth percentile) on the BRB, based on the Italian normative data [5]. Failure on the BICAMS was defined as the failure in at least one test of the battery [10]. Performance on the SDMT was assessed using normative data from the Italian BICAMS validation [10]. The agreement between the performances of the two batteries was assessed through Cohen’s K statistic [14–16]. The sensitivity, specificity and accuracy of the BICAMS against the BRB were assessed. Finally we calculated the effects sizes for each test of the two batteries using Cohen’s d (difference between means divided by pooled SD) separating MS patients and controls [17].

## Results

The study sample consisted of 192 consecutive RRMS patients from 11 Italian MS Centres (Table 1). Table 2 shows mean scores of patients and normative samples on the neuropsychological tests [5, 10]. Mean scores of patients were lower compared to those of healthy subjects in all the cognitive measures examined. To compare the performance on the BICAMS and the BRB, we have repeated the analysis excluding SDMT, which was the only neuropsychological test included in both of the batteries. This exclusion is intended to avoid an overestimation of the accord between the two assessment tools. Forty-six MS patients were identified as impaired and 48 as unimpaired on both batteries, when the SDMT was included in the analysis. Cohen’s K statistic was 0.46 which corresponds to a moderate accord [14–16]. The Cohen’s K statistic estimating the agreement between the SDMT alone and the BRB was comparable (0.42). As expected, the concordance decreased if we excluded the SDMT from the BRB. In this case, 37 MS patients were identified as impaired and 57 as unimpaired on both of the batteries. Cohen’s K statistic was 0,3 (0.26 for the SDMT alone) which corresponds to a poor accord [14–16]. Using the whole BRB as the gold standard, overall BICAMS sensitivity was 58,2 %, specificity 86,7 %, with an accuracy of 75 %. Using the SDMT alone the sensitivity was 43 %, the specificity 95,6 % and accuracy 73,9 %. Table 3 shows Cohen’s d for different tests [17]. Overall, verbal memory tests of the BRB and BICAMS were comparable (SRT-LTS 0.55, SRT-CLTR 0.61, CVLT 2 0.61). Instead, the BVMT-R of the BICAMS showed a higher d value (0.60) as compared with the SPART test (0.38). Finally, the SDMT, PASAT 3 and 2 yielded higher d values (SDMT 0.83, PASAT 3 0.65, PASAT 2 0.84).

**Table 1:** Characteristics of the study sample (# 192 MS subjects)

Age, years, mean (SD)	41.4 (10.8)
Education, years, mean (SD)	12.3 (3.5)
Gender (women, men)	142/50
Disease duration, years, mean (SD)	12.7 (8.9)
EDSS, mean (SD)	2.7 (1.7)
# of relapses in the year prior to inclusion, mean (SD)	0.5 (0.8)
Treatment with DMD, n (%)	154 (80.2)

*MS* multiple sclerosis, *SD* standard deviations, *EDSS* expanded disability status scale, *DMD* disease modifying drugs

**Table 2:** Mean scores (SD) of patients and normative samples on the neuropsychological tests [5, 10]

Test	MS (#192)	HC	*p*
*BRB*			
SRT-LTS	39.9 (14.4)	47.5 (13.1)	<0.001
SRT-CLRT	31.3 (15.0)	40.3 (14.4)	<0.001
SPART	18.9 (5.5)	20.9 (4.9)	<0.001
PASAT-3	36.1 (15.7)	45.0 (10.6)	<0.001
PASAT-2	25.6 (14.1)	36.5 (11.5)	<0.001
SRT-D	7.9 (2.9)	8.9 (2.2)	<0.001
SPART-D	6.4 (2.4)	7.2 (2.4)	0.002
WLG	23.4 (6.7)	26.1 (5.8)	<0.001
*BICAMS*			
SDMT	46.4 (12.8)	56.3 (11.3)	<0.001
CVLT-II	49.9 (12.1)	56.3 (9.0)	<0.001
BVMT-R	23.7 (8.0)	27.9 (6.1)	<0.001

*MS* multiple sclerosis, *HC* healthy control, *BRB* brief repeatable battery, *SRT-LTS* selective reminding test-long term storage, *SRT-CLRT* selective reminding test-consistent long term retrieval, *SPART* spatial recall test, *PASAT-3* paced auditory serial addition test-3 s, *PASAT-2* paced auditory serial addition test-2 s, *SRT-D* selective reminder test-delayed, *SPART-D* spatial recall test-delayed, *WLG* world list generation, *BICAMS* brief international cognitive assessment in multiple sclerosis, *SDMT* symbol digit modalities test, *CVLT-II* California verbal learning test-second version, *BVMT-R* brief visuospatial memory test-revised

**Table 3:** Cohen’s d for each test of the two batteries separating MS patients and controls

Test	d
*BRB*	
SRT-LTS	0.55
SRT-CLTR	0.61
SPART	0.38
PASAT 3	0.65
PASAT 2	0.84
SRT-D	0.38
SPART-D	0.33
WLG	0.43
*BICAMS*	
SDMT	0.83
CVLT-II	0.61
BVMT-R	0.60

*BRB* brief repeatable battery, *SRT-LTS* selective reminding test-long term storage, *SRT-CLRT* selective reminding test-consistent long term retrieval, *SPART* spatial recall test, *PASAT-3* paced auditory serial addition test-3 s, *PASAT-2* paced auditory serial addition test-2 s, *SRT-D* selective reminder test-delayed, *SPART-D* spatial recall test-delayed, *WLG* world list generation, *BICAMS* brief international cognitive assessment in multiple sclerosis, *SDMT* symbol digit modalities test, *CVLT-II* California verbal learning test-second version, *BVMT-R* brief visuospatial memory test-revised

## Discussion

Cognitive assessment represents a key step in taking charge of MS patients. The most widely used neuropsychological battery is BRB [5]. Recently the BICAMS, a rapid tool, more suitable to be incorporated in everyday patient assessment, has been developed [6]. We recently published normative values for the BICAMS in the Italian population [10]. The implementation of BICAMS is still in its infancy; therefore we need to obtain more information about its performance in comparison to other neuropsychological batteries. In the present study we administered to MS patients both the BRB and the BICAMS. Mean scores of patients were lower compared to those of healthy subjects [5, 10] in all the cognitive measures examined. This is in line with the typical cognitive profile in MS patients [1]. In our study, tests with higher discriminating ability according to the d values were the SDMT, PASAT 2 and 3 s. This finding is in line with recent literature, identifying the SDMT as the test with higher ability in differentiating MS patients from healthy controls [18, 19]. On the basis of our results, the agreement between the BICAMS and the BRB is fair to moderate and mainly dependent on the inclusion of the SDMT, which is the only common test between the two batteries. We can hypothesize that the BICAMS and the BRB cannot be considered as equivalent in the assessment of CI in MS. The BRB investigates more comprehensively the cognitive profile, including wider assessment than the BICAMS. Therefore, we can consider the BRB a brief neuropsychological battery more complete than the BICAMS and more suitable to identify change over time [20–22]. The BICAMS, on the other hand, can represent a valid alternative to a more comprehensive battery when available resources are scarce. Since in our study cognitive evaluations were performed by neuropsychologists, further analyses including the administration of tests by health care professionals who are not cognitive specialists are needed, in order to confirm the external validity of our findings.

## Conclusions

In conclusion, on the basis of our results, we can consider the BICAMS a brief and feasible tool appropriate for the cognitive assessment of MS patients and for research use. It seems important to stress that, in optimal clinical conditions, where a neuropsychologist can take care of the cognitive assessment, it is preferable to achieve a more thorough cognitive evaluation using tools including neuropsychological tests that investigate several cognitive domains impaired in MS.

## Abbreviations

BICAMS: Brief international cognitive assessment for multiple sclerosis
MS: Multiple sclerosis
BRB: Brief repeatable battery
CI: Cognitive impairment
SDMT: Symbol digit modalities test
PASAT 3 and 2: Paced auditory serial addition test 3 and 2
RR: Relapsing-remitting
SRT: Selective reminding test
SPART: 10/36 spatial recall test
WLG: Word list generation
CVLT-II: California verbal learning test, second edition
BVMT-R: Brief visuospatial memory test-revised
